# Reply to Perrella et al. Coming Back to the Basics. Comment on “Cangir et al. A CT-Based Radiomic Signature for the Differentiation of Pulmonary Hamartomas from Carcinoid Tumors. *Diagnostics* 2022, *12*, 416”

**DOI:** 10.3390/diagnostics13233490

**Published:** 2023-11-21

**Authors:** Ayten Kayi Cangir, Kaan Orhan, Aysegul Gursoy Coruh

**Affiliations:** 1 Department of Thoracic Surgery Ankara, Ankara University Faculty of Medicine (AUFM), Ankara 06100, Turkey; 2 Medical Design Application and Research Center (MEDITAM), Ankara University, Ankara 06100, Turkey; call53@yahoo.com; 3 Department of Dental and Maxillofacial Radiodiagnostics, Medical University of Lublin, 20-093 Lublin, Poland; 4 Department of Dentomaxillofacial Radiology, Ankara University Faculty of Dentistry, Ankara 06100, Turkey; 5 Department of Radiology, Ankara University Faculty of Medicine (AUFM), Ankara 06100, Turkey; coruh@medicine.ankara.edu.tr

We thank to Dr. Perrella and and his fellow authors for your kind letter and thoughtful comments [1] about our article [2]. It is important to distinguish between pulmonary hamartomas (PHS) and carcinoid tumors, which require very different clinical forms of management. When located peripherally, the differentiation of a carcinoid from a hamartoma becomes more difficult. It is estimated that nearly half of the carcinoids are located peripherally [3,4]. Differentiating carcinoids from hamartomas is essential because carcinoids may have aggressive behaviors. Besides the fat attenuation in the hamartoma, there are several other computed tomography (CT) features like the presence of hyper-attenuated area, atelectasis or post-obstructive nodules distal to the neoplasm, which are more commonly observed in carcinoid tumors [3,4,5,6]. In addition to those findings, we also defined a new sign in our study, which we have called the ‘bronchial triangular sign’ [3]. It shows the extension of the carcinoid tumor along the bronchial wall on CT and is thought to represent the infiltration of the bronchial wall and the invasion of the parenchyma pathologically. In that study, 84% of the carcinoids, that had no detectable endo-bronchial components on CT, showed the bronchial triangular sign. We also compared the attenuation values on contrast-enhanced CTs. We showed that carcinoids had higher median attenuation values compared to hamartomas (*p* < 0.001). In our study, carcinoids showed a median attenuation value of 84 HU (min–max; 32–172 HU) and hamartomas had a median attenuation value of 22.5 HU (min–max; −123–71 HU) on contrast-enhanced CT. In addition to these findings, as you mentioned, the wash-out ratios might help in the differentiation. But the important limitation of your study is the increased radiation dose of multiphasic study compared to a single-phase CT. In clinical practice, by using multiphasic evaluation, the effective radiation dose increases and it cannot be routinely used for nodule screening, which is not compatible with ALARA (as low as reasonably achievable) principles. Nevertheless, the cut-off wash-out ratios, which are defined in larger cohorts with high sensitivity and specificity values, might be used for the specific patients who have pulmonary carcinoid suspension.

On the other hand, in our study, CT radiomic features are evaluated to differentiate PHs from pulmonary carcinoid tumors (PCTs). A total of 138 patients (78 PCTs and 60 PHs) were evaluated. Two handcrafted radiomics models are prepared in this study: the first model includes the data of all patients to be differentiated between the groups; the second model includes 78 PCTs and 38 PHs without signs of fat tissue. The separation of the training and validation datasets was performed randomly using an 8:2 ratio and 620 random seeds. The results revealed that the MLP method (RF) was best for PH (AUC = 0.999) and PCT (AUC = 0.999) for the first model (AUC = 0.836), and PC (AUC = 0.836) in the test set for the second model. Radiomics tumor features derived from CT images are useful to differentiate the carcinoid tumors from hamartomas with high accuracy. Radiomics features may be used to differentiate PHs from PCTs with high levels of accuracy, even without the presence of fat on the CT. 

Conclusions: Bagnacci et al.’s comment was not seen as an attempt to belittle our work, because in science, there may be different solutions to problems. Our study was conducted with data from 138 patients and without additional X-ray exposure to patients. We hope you will find our response satisfactory and thank you once more for your comments.

## References

[1] Perrella A., Bagnacci G., Di Meglio N., Di Martino V., Bellan C., Luzzi L., Mazzei M.A., Volterrani L. (2023). Coming Back to the Basics. Comment on Cangir et al. A CT-Based Radiomic Signature for the Differentiation of Pulmonary Hamartomas from Carcinoid Tumors. *Diagnostics* 2022, *12*, 416. Diagnostics.

[2] Cangir A.K., Orhan K., Kahya Y., Uğurum Yücemen A., Aktürk İ., Ozakinci H., Gursoy Coruh A., Dizbay Sak S. (2022). A CT-Based Radiomic Signature for the Differentiation of Pulmonary Hamartomas from Carcinoid Tumors. Diagnostics.

[3] Meisinger Q.C., Klein J.S., Butnor K.J., Gentchos G., Leavitt B.J. (2011). CT features of peripheral pulmonary carcinoid tumors. AJR Am. J. Roentgenol..

[4] Coruh A.G., Kul M., Öz D.K., Yenigün B., Cansız Ersöz C., Özalp Ateş F., Atasoy Ç. (2020). Is it possible to discriminate pulmonary carcinoids from hamartomas based on CT features?. Clin. Imaging..

[5] Gleeson T., Thiessen R., Hannigan A., Murphy D., English J.C., Mayo J.R. (2013). Pulmonary hamartomas: CT pixel analysis for fat attenuation using radiologic-pathologic correlation. J. Med. Imaging Radiat. Oncol..

[6] Jeung M.Y., Gasser B., Gangi A., Charneau D., Ducroq X., Kessler R., Quoix E., Roy C. (2002). Bronchial carcinoid tumors of the thorax: Spectrum of radiologic findings. Radiographics.

